# Immune-based treatment re-challenge in renal cell carcinoma: A systematic review and meta-analysis

**DOI:** 10.3389/fonc.2022.996553

**Published:** 2022-12-02

**Authors:** Maria Papathanassiou, Ioannis Tamposis, Kalliopi K. Exarchou-Kouveli, Panagiota I. Kontou, Anna Tzortzi de Paz, Lampros Mitrakas, Maria Samara, Pantelis G. Bagos, Vassilios Tzortzis, Panagiotis J. Vlachostergios

**Affiliations:** ^1^ Department of Pathology, Faculty of Medicine, School of Health Sciences, University of Thessaly, Larissa, Greece; ^2^ Department of Computer Science and Biomedical Informatics, University of Thessaly, Lamia, Greece; ^3^ Department of Mathematics, University of Thessaly, Lamia, Greece; ^4^ Department of Urology, Faculty of Medicine, School of Health Sciences, University of Thessaly, University Hospital of Larissa, Larissa, Greece; ^5^ Department of Medicine, Division of Hematology and Medical Oncology, Weill Cornell Medicine, New York, NY, United States

**Keywords:** immunotherapy, immune checkpoint inhibitor, rechallenge, salvage, second-line, VEGF TKI, renal cell carcinoma

## Abstract

**Introduction:**

The use of immune checkpoint inhibitors (ICIs) as a front-line treatment for metastatic renal cell carcinoma (RCC) has significantly improved patient’ outcome. However, little is known about the efficacy or lack thereof of immunotherapy after prior use of anti-PD1/PD-L1 or/and anti-CTLA monoclonal antibodies.

**Methods:**

Electronic databases, including PubMed, EMBASE, Medline, Web of Science, and Cochrane Library, were comprehensively searched from inception to July 2022. Objective response rates (ORR), progression-free survival (PFS), and ≥ grade 3 adverse events (AEs) were assessed in the meta-analysis, along with corresponding 95% confidence intervals (CIs) and publication bias.

**Results:**

Ten studies which contained a total of 500 patients were included. The pooled ORR was 19% (95% CI: 10, 31), and PFS was 5.6 months (95% CI: 4.1, 7.8). There were ≥ grade 3 AEs noted in 25% of patients (95% CI: 14, 37).

**Conclusion:**

This meta-analysis on different second-line ICI-containing therapies in ICI-pretreated mRCC patients supports a modest efficacy and tolerable toxicity.

## Introduction

Renal cell carcinoma (RCC) is a commonly diagnosed urological malignancy with rising incidence rates (1). Despite decreasing mortality rates in developed countries, advanced RCC remains lethal and thus further progress in the current therapeutic armamentarium and sequencing of systemic therapies is needed. Clear-cell RCC comprises 75% of RCC cases (2, 3).

Until recently, standard first-line treatment therapies for metastatic clear cell renal cancer (mRCC) have been mostly targeted against signaling through the vascular endothelial growth factor receptor (VEGFR), either *via* use of tyrosine kinase inhibitors (TKIs) such as sunitinib and pazopanib (4, 5), or monoclonal antibodies i.e. bevazicumab (6). Patients with disease progression after treatment with first-line anti-angiogenic agents (AA), were destined to receive another VEGFR TKI or/and mTOR inhibitor (7).

Immune checkpoint inhibitors (ICIs) have revolutionized the treatment landscape of RCC, initially at second-line with superiority of nivolumab over everolimus in the CheckMate 025 study (8) and most recently in the first-line setting with ICI-ICI and ICI-VEGFR TKI combinations (9–12). ICIs approved in advanced RCC are monoclonal antibodies against immune checkpoints including the programmed cell death protein 1 (PD-1) or its ligand (PD-L1) and CTLA-4 (13). The binding of cancer cells to immune cells through these checkpoints leads to immune response downregulation and subsequent cytokine release inhibition which, in turn reduces the cytotoxic T-cell activity against tumors (13). This process is reversed by ICIs.

The expanded use of immunotherapy and VEGFR TKIs (ICI-ICI and ICI-VEGFR TKI combinations) in the front-line setting is changing the landscape of subsequent therapies as well. As a result, choosing between available beyond first-line options upon progression has become more challenging. In this context, it remains elusive whether a re-challenging approach, particularly with respect to ICIs could lead to clinically meaningful responses in later lines of therapy in patients with metastatic RCC. In this systematic review and meta-analysis, we provide insight to the efficacy and safety of immunotherapy as a second-line treatment in patients with mRCC who were previously treated with ICIs.

## Materials and methods

### Eligibility criteria

This study developed the inclusion and exclusion criteria based on “PICOS” principles. Inclusion criteria were as follows: (i) Design of studies, prospective, retrospective or ambispective; (ii) patients (P), patients with metastatic RCC who received at least one prior line of systemic therapy that included an immune checkpoint inhibitor; (iii) intervention (I), second-line immune checkpoint inhibitor; (iv) control (C), not-applicable; (v) outcomes (O), the primary endpoints were objective response rate (ORR), which was defined as percentage of complete (tumor disappearance), or partial (tumor shrinkage ≥ 30%) decrease in the baseline sum of the longest diameter of target lesions and progression-free survival (PFS), which was defined as the length of time that patients lived with the tumor without evidence of progression. The secondary endpoint was ≥ grade 3 toxicity, which according to the Common Terminology Criteria for Adverse Events (CTCAE) was defined as severe or medically significant but not immediately life-threatening adverse events or resulting in hospitalization or prolongation of hospitalization indicated, disabling or limiting self-care activities of daily living (ADL).

### Search methodology

The selection and systematic review of clinical studies were performed and reported in accordance with the Preferred Reporting Items for Systematic Reviews and Meta-Analyses (PRISMA) statement (14). The search was limited to studies published in English. We searched PubMed, the Cochrane Library, EMBASE and Web of Science electronic database. Eligible studies were obtained, using search terms (i) renal OR kidney; (ii) cancer OR carcinoma OR tumor OR neoplasm; (iii) renal OR kidney AND cancer OR carcinoma OR tumor OR neoplasm; (iv) metastases OR metastatic; (v) renal OR kidney AND cancer OR carcinoma OR tumor OR neoplasm AND metastases OR metastatic; (vi) salvage OR second-line; (vi) immunotherapy OR immune checkpoint inhibitor; (vii) renal OR kidney AND cancer OR carcinoma OR tumor OR neoplasm AND metastases OR metastatic AND salvage OR second-line AND immunotherapy OR immune checkpoint inhibitor. We included studies up until July 2022. A manual screen of study references was also conducted to obtain possibly relevant literature. After excluding repeated studies, we screened all articles based on their title, abstract, and full text.

### Data extraction

Using a standardized data extraction form, two investigators independently extracted the following data from each study: (i) Study ID, including the name of the first author and publication year; (ii) country where the study was performed; (iii) study subjects, number of participants and their ages; (iv) treatment regimens; and (v) treatment outcomes, including objective response rate (ORR), progression-free survival (PFS), and ≥ Grade 3 toxicity. For reports of the same study at different follow-up periods, data from the last report were used for analysis.

### Statistical analysis

Based on the data available from the studies we analyzed the Objective Response Rate (ORR), the median progression-free survival (PFS) and the proportion of patients with ≥3 Grade AEs and. ORR and the proportion of patients with Grade ≥3 AEs needed methods suitable for rates and proportions. We used the statistical software Stata, with the Freeman-Tukey double arcsine transformation implemented in metaprop (15) and metan and the logit transformation (16) implemented with metan. For PFS we used its logarithm along with 95% confidence intervals provided by the studies (17). In all cases we used the inverse-variance random-effects method of DerSimonian and Laird (18) in order to account for between studies variability (heterogeneity). The I-squared index was used to quantify heterogeneity. Publication bias was estimated using the Egger regression test (19) and the Begg’s and Mazumbar’s rank correlation test (20).

## Results

### Study selection outcome

**Figure 1:**
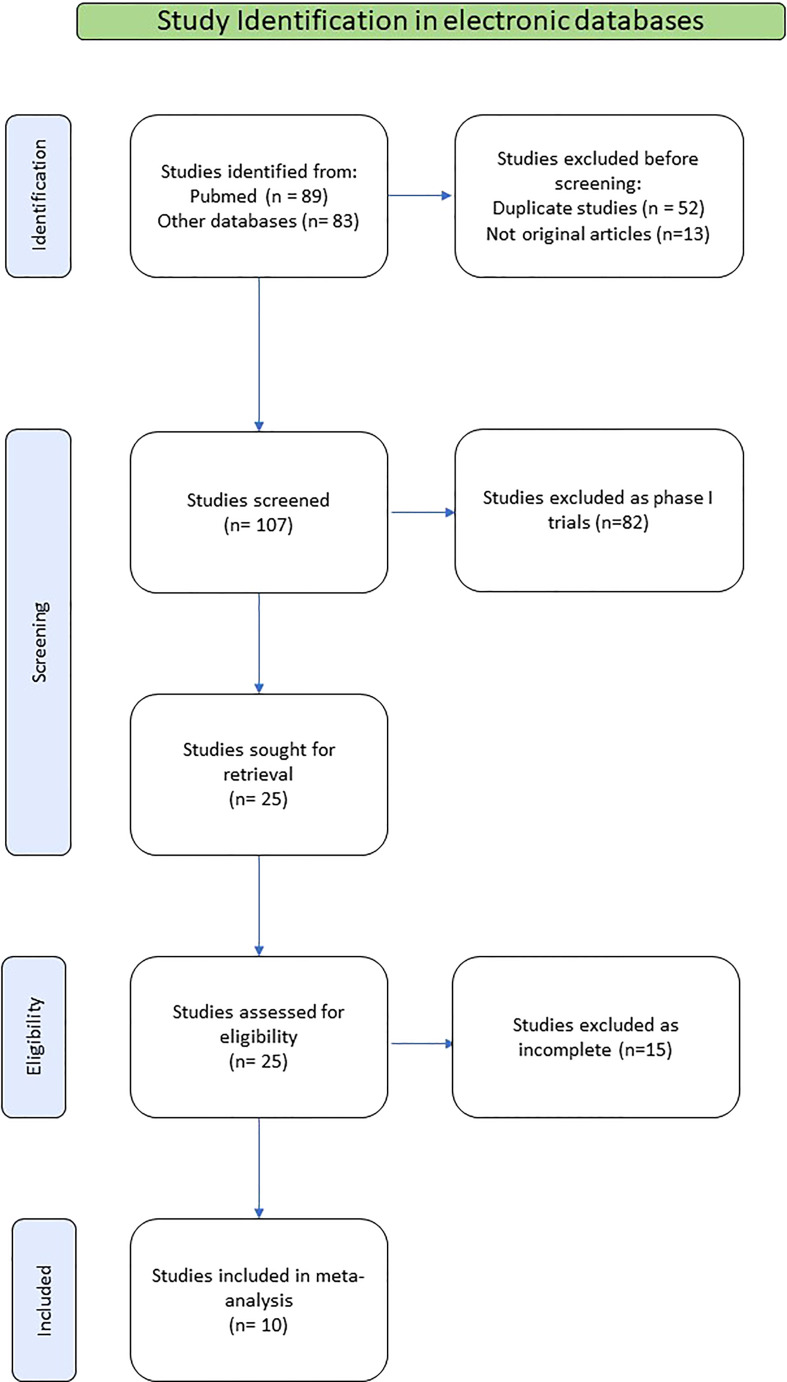
Flowchart of the selection process for eligible studies.

Among the publications retrieved using electronic search (N=89), 10 studies were eligible for the present meta-analysis, including a total of 500 patients (21–30). The detailed flowchart of the selection process for eligible studies is depicted in **Figure 1**.

### Study characteristics

The studies included in this meta-analysis were published between 2020 and 2022. With regards to treatment, 7 studies used nivolumab plus ipilimumab, or nivolumab alone as second-line therapy (21, 23–28), one study used the combination of pembrolizumab and lenvatinib (29) and another used the combination of atezolizumab and bevacizumab (30). A multicenter retrospective cohort study analyzed various combinations including nivolumab/ipilimumab, pembrolizumab/axitinib, pembrolizumab/bevacizumab, atezolizumab/investigational agent, nivolumab/investigational agent, avelumab/chemotherapy, spartazilumab/investigational agent, and monotherapies with pembrolizumab, nivolumab, or durvalumab (22). All studies reported ORR and PFS as outcomes as well as safety data. The clinical characteristics of the included studies are presented in **Table 1**.

**Table 1:** Characteristics of included studies.

Study (author,year)	Design	N	Age (medianl	IMDC(%) fav/int/poor	Prior line	2^nd^ line	N of cycles	ORR(%)	PFS (mos)	Grade 3 M.s (%)
Gul et a l. 2020 (21)	retros pective	45	62	20/64/7	ICI± other	Nivo+lpi	≥1	9/45 (20%)	4(0.8-19)	6/45(13%)
Ravi et al. 2020 (22)	retrospective	69	61	19/65/12	ICI±ICI or ICI+AA	ICI±ICI or ICI+AA	2-8	15/64 (23%)	5.7(3.2- 7.6)	11/69 (16%)
Choueiri et al. 2020 FRACllON-RCC) (23)	Phase II	46	NA	NA	ICI AA (80%)	Nivo+lpi	≥1	7/46 (15.2%)	4 (2.3-7.9)	13/46(28.3%)
McK ay et al. 2020 (OMNIVOR E) (24)	Phase II	57	63	34.1/5 6.8 /9.	Nivo	Nivo+lpi	1-2	2/57 (4%)	4.7 (2.7- 8.3)	14/57 (25%)
Grimm et a l. 2021(TITAN- RCC) (25)	Phase II	28	65	0/71/25	Nivo	Nivo+lpi	24	3/28 (11%)	3.7 (24.5)	NA
Atkins et al. 2022 (HCRN GU16-260) (26)	Phase II	35	65	17.2/77.1/5.7	Nivo	Nivo+lpi => Nivo	4	4/3 5 (11.4%)	8.3( 5.5- 10.9	15/35 (42.9%)
Yang et al. 2021(27 I	retrospective	27	61.4	6/13/ 5	Nivo	Nivo+lpi => Nivo	≥1	5/22 (23%)	4(2.4-6.2)	5/27(18.5)
Vauchier et al. 2022 (28)	ambispective	45	59	23/25/53	ICI±ICI, ICI+AA	Nivo±lpi	≥1	7/45 (16%)	3.5 (2.8- 9.7)	2/45 (4%)
Lee et al. 2021(KEYNOTE-146) (29)	Phase lb/11	104	60	17/59/24	ICI±ICI, ICI+AA	Pembr o+L enva	8	58/104 (55.8%)	12.2 (9.5- 17.7)	59/104(57%)
Powles et a l. 2021 (1Mmotion150) (30)	Phase II	44	61	NA	Atezo	Atezo+Beva	≥1	11/44 (25%)	8.7( 5.6- 13.7)	NA (for the entire study N=103, 31/103 (30%)

### ORR

**Figure 2:**
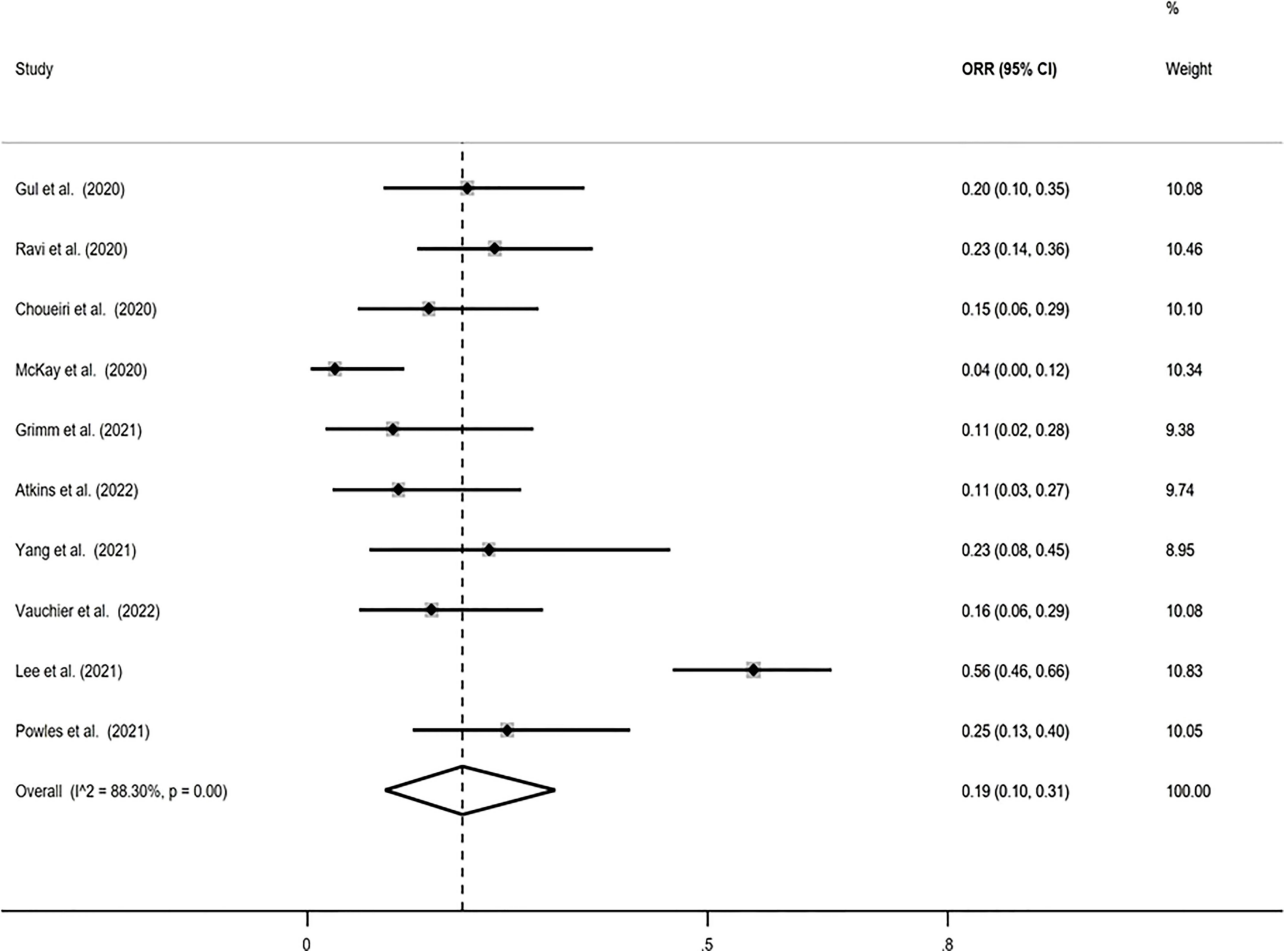
Forest plot displaying the pooled objective response rate (ORR) proportion in random-effects meta-analysis with the Freeman-Tukey double arcsine transformation.

The pooled ORR using the Freeman-Tukey double arcsine transformation was calculated equal to 0.19 (95% CI: 0.10, 0.31), with I-squared equal to 88.30%. Similar estimates were obtained with the logit transformation, ORR=0.19 (95% CI: 0.11, 0.31) with I-squared=85.9% (**Figure 2**).

### PFS

**Figure 3:**
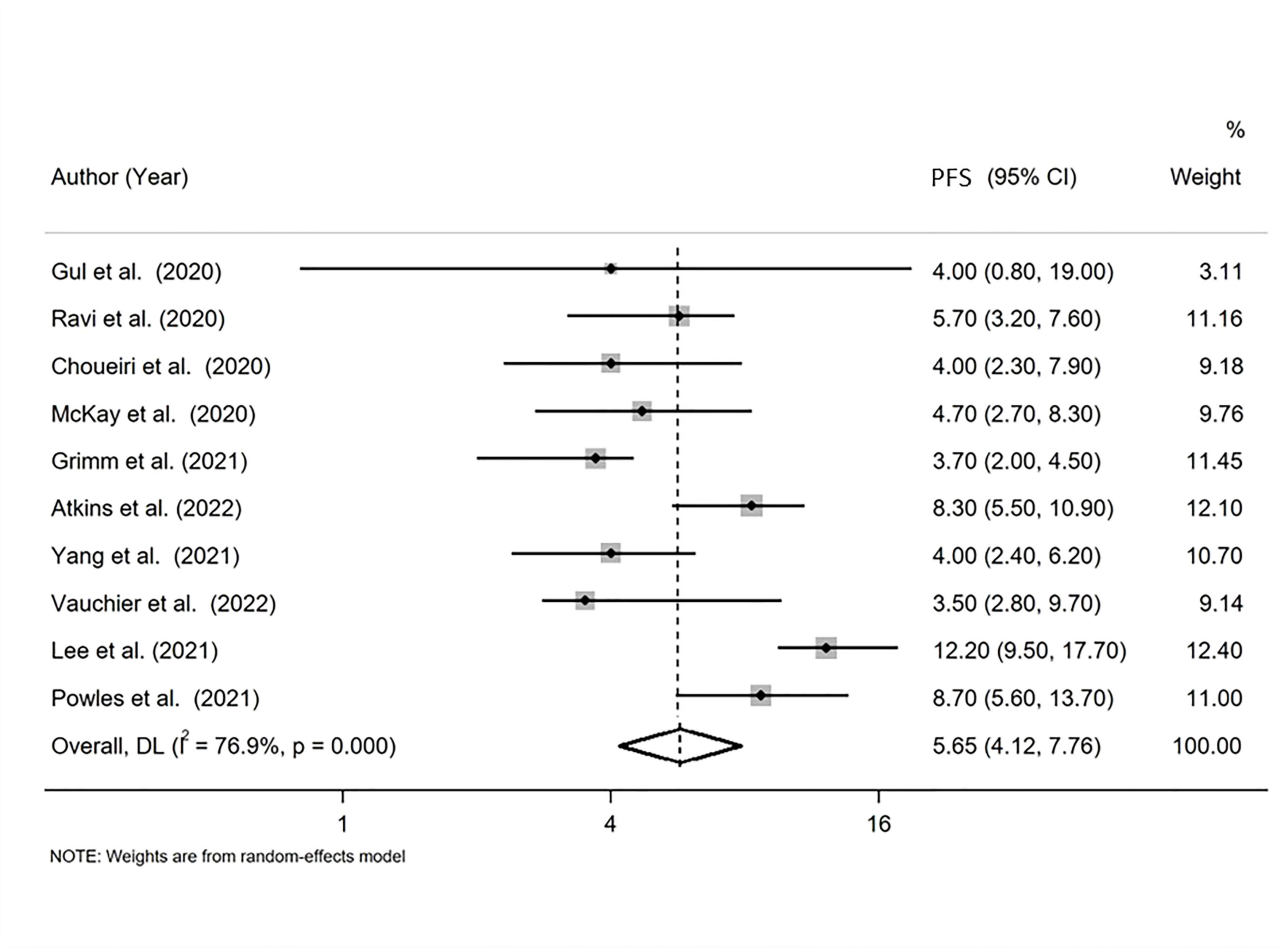
Forest plot displaying the pooled median progression-free survival (PFS) in random-effects meta-analysis.

The pooled PFS was found equal to 5.655 months (95% CI: 4.120, 7.762 months) with I-squared equal to 76.9% (**Figure 3**).

### Serious AEs

**Figure 4:**
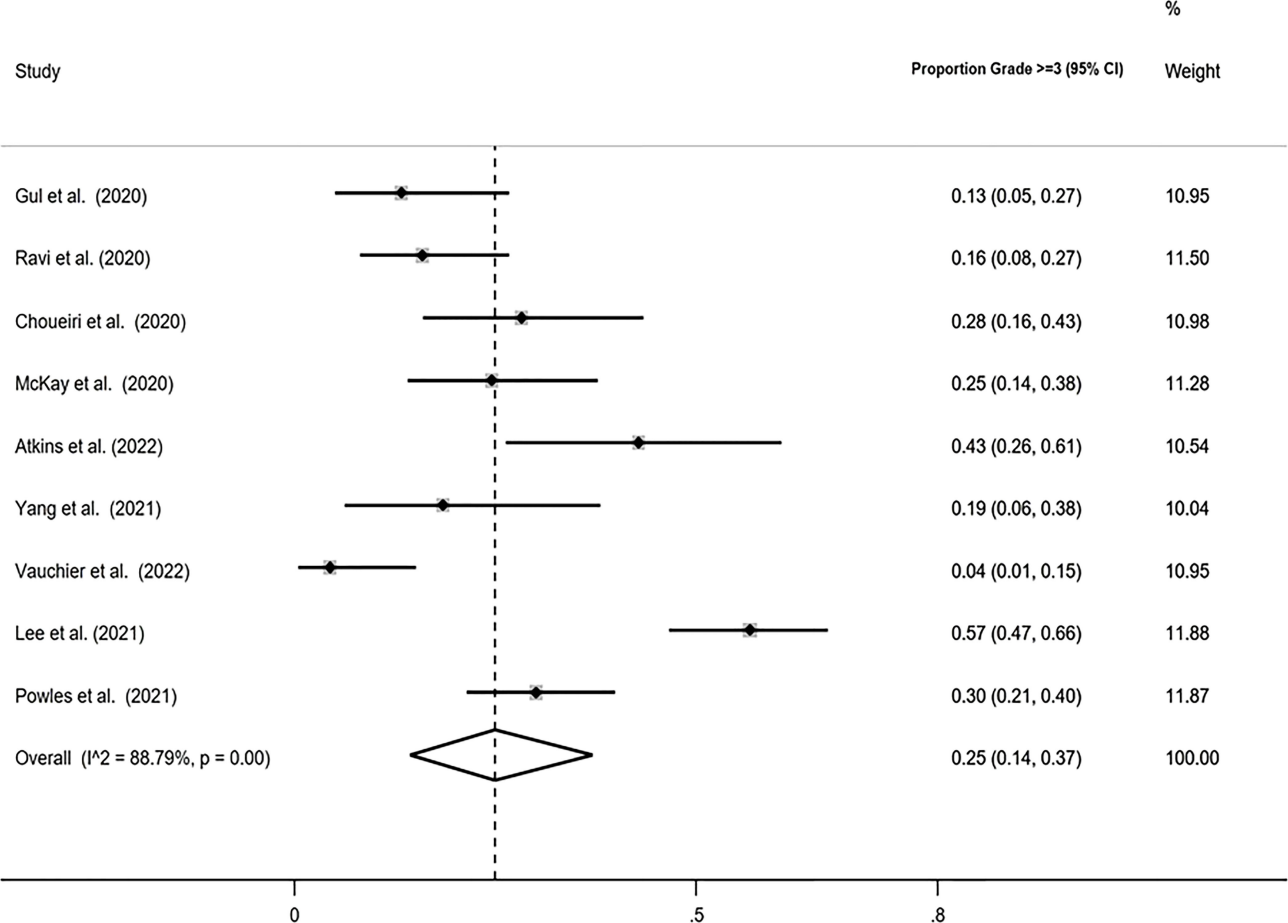
Forest plot displaying the pooled Grade ≥3 proportion in random-effects meta-analysis with the Freeman-Tukey double arcsine transformation.

The pooled proportion of patients with Grade ≥3 AEs using the Freeman-Tukey double arcsine transformation was calculated equal to 0.25 (95% CI: 0.14, 0.37), with I-squared equal to 88.79%. Similar estimates were obtained with the logit transformation, ORR=0.25 (95% CI: 0.16, 0.37) with I-squared=86% (**Figure 4**).

### Publications bias

**Figure 5:**
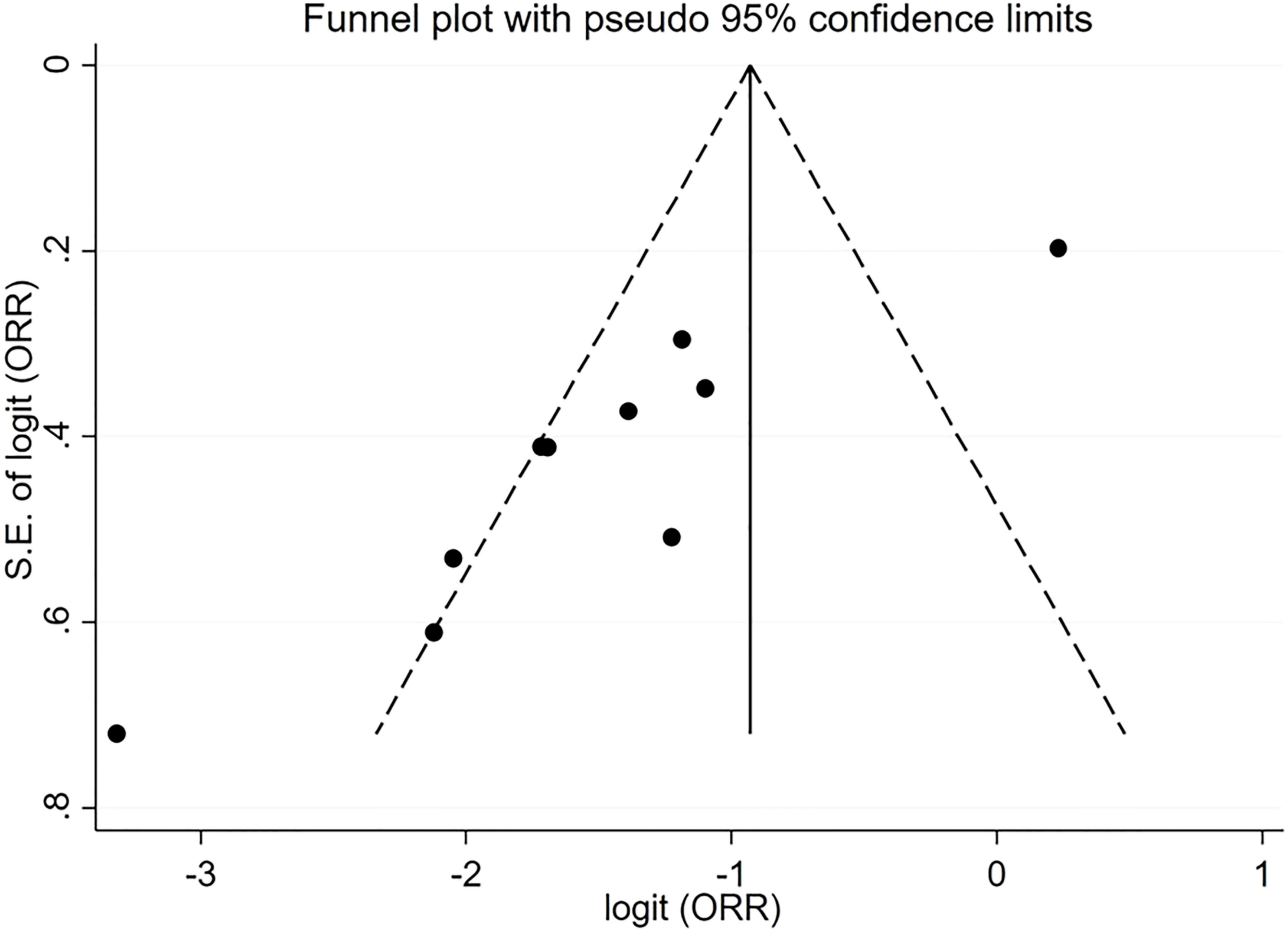
Funnel plot with pseudo 95% confidence limits for the estimation of the publication bias for the objective response rate (ORR) proportion with the logit transformation.

**Figure 6:**
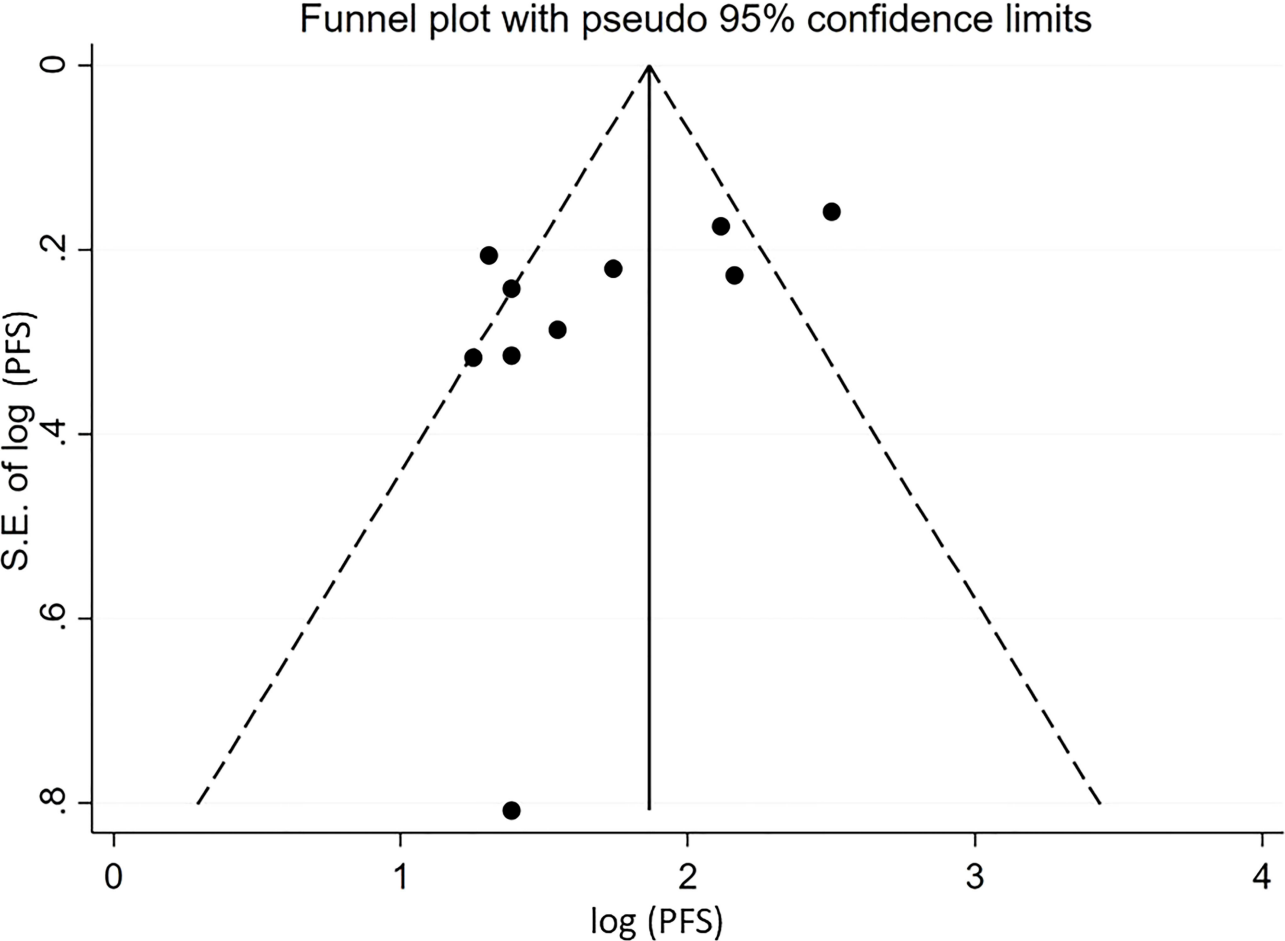
Funnel plot with pseudo 95% confidence limits for the estimation of the publication bias for the median progression-free survival (PFS) with the logarithm transformation.

**Figure 7:**
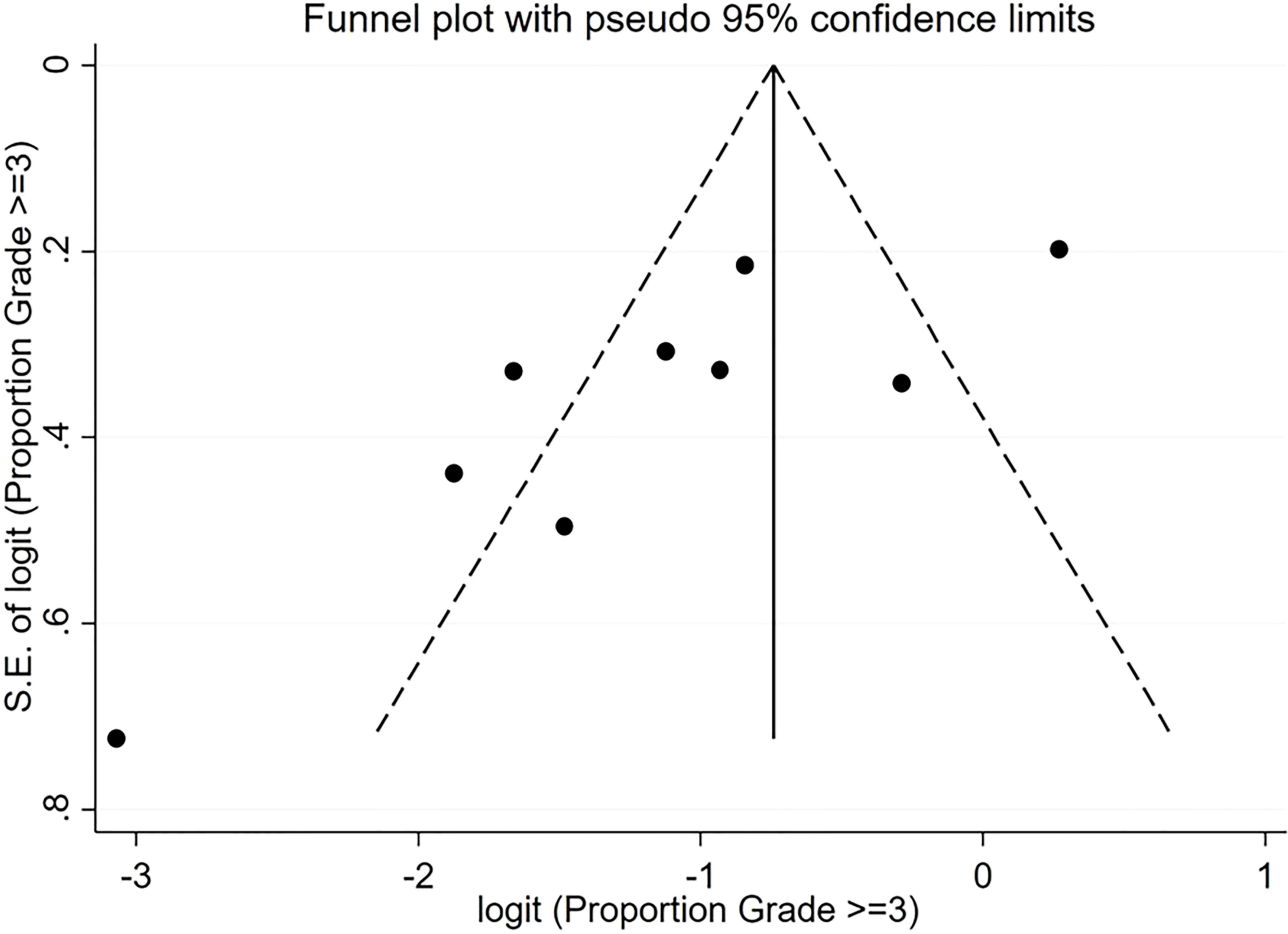
Funnel plot with pseudo 95% confidence limits for the estimation of the publication bias for the Grade ≥3 proportion with the logit transformation.

For pooled ORR analysis, both tests for publication bias, including Egger’s and Begg’s, suggested the presence of it (p-value<0.0001 and 0.012 respectively) (**Figure 5**). With respect to pooled PFS, neither test suggested any evidence of it (p-value=0.078 and 0.283 respectively) (**Figure 6**). Regarding the pooled proportion of patients with Grade ≥3 toxicity, both tests for publication bias suggested the presence of it (p-value=0.016 and 0.048 respectively) (**Figure 7**).

## Discussion

This systematic review and meta-analysis were conducted to answer the question of whether ICI rechallenging in patients with mRCC who have progressed after anti-PD-1/PD-L1 as part of front-line therapy is a safe approach that could result in clinically meaningful responses. Our study showed an ORR of 19% for beyond first-line ICI treatment combinations, mostly including nivolumab/ipilimumab, lenvatinib/pembrolizumab, atezolizumab/bevacizumab and to a lesser extent other ICI/VEGFR TKI combinations. Pooled PFS was 5.655 months and grade ≥3 adverse events were experienced by one quarter of patients (25%).

The synthesis of this meta-analysis involved a heterogenous group of phase II prospective trials with adaptive or fixed design, retrospective studies and a control study with varying sample sizes, first-line treatments and number of previous lines. Among the included studies, three phase II non randomized trials evaluated salvage therapy with nivolumab plus ipilimumab in patients who had received nivolumab monotherapy as first-line treatment (HCRNGU16-260, TITAN-RCC, OMNIVORE) (24–26) and were non-responders. The lowest ORR was observed in the OMNIVORE trial (4%), which might be attributed to patients receiving only 1-2 cycles of combination second-line therapy, whereas, the other two trials administered 2-4 cycles. Overall, ICI monotherapy followed by salvage ICI combination did not achieve good responses neither in the first- nor in the second-line settings.

In the study of Ravi et al. (22) which included various ICI/ICI and ICI/VEGFR TKI combinations, higher ORR at second-line was observed in patients who responded in first-line, compared to those who progressed or had stable disease in the first-line, but remained similar to those receiving first-line monotherapy, suggesting that responses can be observed in second-line immunotherapy and that resistance can be overcome when using different ICIs combined with VEGFR TKIs (22). Similar and higher ORRs were noted in the other two trials that tested an ICI/VEGFR TKI combination beyond first-line (29, 30). In the pembrolizumab-lenvatinib study of Lee et al. (29), more than half (56%) of patients responded despite the fact that two-thirds (65%) of patients had already received a TKI as part of first-line combination therapy, while in the atezolizumab-bevacizumab study of Powles et al. (30) a quarter (25%) of all VEGFR inhibition-naïve patients responded This could imply a sensitizing effect of VEFGR pathway inhibition to further ICI or/and an immune-independent way of completely avoiding cross-resistance particularly in VEGFR TKI-naïve patients. Another important observation across different studies is that the poorest responders to beyond first-line combinations included those with a high burden of metastases (≥1-3), presence of brain metastatic sites and deteriorated ECOG performance status (≥ 2) (21, 28). In this patient population, the ICI/VEGFR TKI combination seemed to be more active if indirectly compared to double ICI, judging from the high ORR (55.8%) and prolonged PFS (12.5 months) of the pembrolizumab/lenvatinib regimen (29).

The results of this meta-analysis are in line with two previous meta-analyses that examined the activity of salvage nivolumab/ipilimumab after prior PD-1 blockade with nivolumab (31, 32). They reported a pooled ORR of 10% (31) and 14% (32), respectively, while PFS ranged between 3.7 and 5.5 months (32). Our study further complements these two meta-analyses by additionally providing a more comprehensive landscape of how ICI works beyond first-line overall, either as ICI doublet or as ICI/VEGFR TKI combination, particularly having also included the studies of Lee et al. (29) and Powles et al. (30), as well as updated data from previous nivolumab/ipilimumab studies.

There were no new safety signals, and all three meta-analyses, including ours reported a comparable percentage of pool incidences of ≥ grade 3 events of 25%-26% (27, 31).

Two additional retrospective studies focusing solely on ICI/TKI combinations reported relatively high objective response (51% and 37.5% respectively) and median PFS (11.6 and 14.2 months in the second-line setting (32, 33). These two studies were excluded from our meta-analysis due to high inherent heterogeneity with respect to including a heavily pre-treated population with at least 2 prior lines of therapy (32) less than half of whom had received ICI during first-line therapy (32, 33).

Because this meta-analysis aimed to explore as many ICI-inclusive options as possible in beyond first-line treatment of mRCC, variations in the types and duration of administration of treatment regimens used, inconsistent timing between anti-PD-1/PD-L1 failure and salvage ICI-containing second-line therapy among these studies, inconsistent baseline clinical data, including IMDC and MSKCC prognostic groups, were inevitable and may have all resulted in the heterogeneity observed. Another limitation of this analysis is derived from the inherent sparseness of ICI-rechallenge studies in mRCC. A greater number of prospective clinical trials with more homogenous inclusion criteria, treatment design and longer follow up would help minimize heterogeneity among studies, and provide a clearer picture on these patients’ outcomes. Individual data could also provide a clearer image on the putative correlation between first and subsequent lines of treatment with ICIs for eligible patients. All data was retrieved directly from publications. Additionally, one of the studies was only available in abstract form; however, it was included due to its unique design. This fact, along with a publication bias calculated in the logit scale, indicate that results should be interpreted with caution.

This meta-analysis on different second-line ICI combinations in ICI-pretreated mRCC patients supports a modest efficacy and tolerable toxicity. A careful selection of the subset of ICI-pretreated patients who are most likely to benefit from ICI-containing therapies beyond first-line should take place for treatment decision-making. Phase III randomized trials of various ICI-TKI combinations after prior ICI are currently ongoing (NCT04987203, NCT04338269, NCT03793166). For example, atezolizumab combined with cabozantinib is currently being tested in the pivotal, global phase III CONTACT-03 trial in patients with inoperable, locally advanced or metastatic renal cell carcinoma (RCC) who progressed during or following treatment with an ICI (NCT04338269). The concept of ICI rechallenge after progression is expanding in other primaries. Although randomized comparisons are lacking, preliminary evidence from individual cases (34, 35) and metaanalyses (36, 37) support its safety with low to modest efficacy, e.g. 8-13% ORR in non-small cell lung cancer, depending on the clinical context.

## Author contributions

PV, VT, and PB conceptualized and designed the study. PV, MP, and IT performed the search and drafted the manuscript. MP and PV performed the data extraction. KE-K, PK, and PB analyzed the data. IT, KE-K, PK, and PB provided the clinical imaging data of the patients. MP, LM, MS, PB, VT, and PV reviewed and revised the original draft. All authors contributed to the article and approved the submitted version.
